# The effect of high count rates on cardiac perfusion quantification in a simultaneous PET-MR system using a cardiac perfusion phantom

**DOI:** 10.1186/s40658-017-0199-y

**Published:** 2017-12-11

**Authors:** Jim O’ Doherty, Zacharias Chalampalakis, Paul Schleyer, Muhummad Sohaib Nazir, Amedeo Chiribiri, Paul K. Marsden

**Affiliations:** 1 PET Imaging Centre, Division of Imaging Sciences and Biomedical Engineering, King’s College London, St. Thomas’ Hospital, London, UK; 2 Department of Molecular Imaging, Sidra Medical and Research Center, Al Luqta St, Doha, Qatar; 3 Siemens Healthineers UK, Frimley, Camberley UK; 4 BHF Centre of Excellence, NIHR Biomedical Research Centre and Wellcome Trust and EPSRC Medical Engineering Centre at Guy’s and St. Thomas’ NHS Foundation Trust, London, UK; 5 Department of Cardiology, Guy’s and St Thomas’ NHS Foundation Trust, London, UK

**Keywords:** PET-MR, Cardiac PET, Dead time, Perfusion

## Abstract

**Background:**

PET-MRI is under investigation as a new strategy for quantitative myocardial perfusion imaging. Consideration is required as to the maximum scanner count rate in order to limit dead-time losses resulting from administered activity in the scanner field of view during the first pass of the radiotracer.

**Results:**

We performed a decaying-source experiment to investigate the high count-rate performance of a PET-MR system (Siemens mMR) over the expected range of activities during a clinical study. We also performed imaging of a cardiac perfusion phantom, which provides an experimental simulation of clinical transit of a simultaneous radiotracer (phantom injected activities range 252 to 997 MBq) and gadolinium-based contrast agent (GBCA). Time-activity and time-intensity curves of the aorta and myocardium compartments from PET and MR images were determined, and quantification of perfusion was then performed using a standard cardiac kinetic model. The decaying-source experiment showed a maximum noise equivalent count rate (NECR_max_) of 286 kcps at a singles rate of 47.1 Mcps. NECR was maintained within 5% (NECR_95%_) of the NECR_max_ with a singles rate of 34.1 Mcps, corresponding to 310 MBq in the phantom. Count-rate performance was degraded above the singles rate of 64.9 Mcps due to the number of detection events impacting the quantitative accuracy of reconstructed images. A 10% bias in image activity concentration was observed between singles rates of 78.2 and 82.9 Mcps. Perfusion phantom experiments showed that image-based activity concentration and quantified values of perfusion were affected by count losses when the total singles rate was greater than 64.9 Mcps. This occurred during the peak arterial input function (AIF) phase of imaging for injected activities to the phantom of 600 MBq and greater.

**Conclusions:**

Care should be taken to avoid high count-rate losses in simultaneous PET-MRI studies. Based on our results in phantoms, bias in reconstructed images should be avoided by adhering to a singles rate lower than 64.9 Mcps on the mMR system. Quantification of perfusion values using singles rates higher than 64.9 Mcps on this system may be compromised and should be avoided.

## Background

The availability of hybrid PET-MRI scanners gives the ability to perform simultaneous PET-MR cardiac studies, combining the functional perfusion quantitation from PET with excellent soft tissue contrast and high spatial and temporal resolution of cardiac MRI. Although the technique of simultaneous PET-MR myocardial perfusion imaging (MPI) studies is still in its infancy, multiple simultaneous image sets of structure and function of the same area of tissue may aid the clinician in the understanding of the pathophysiology of a wide range of cardiovascular diseases.

In order to provide reliable quantification of perfusion using PET, technical requirements should be considered, such as the presence of respiratory or motion artifacts [1, 2], analysis software for calculating perfusion [3, 4], prompt gamma correction [5] and image reconstruction [6]. A further subtle requirement is an upper limit on the injected activity in order to limit the count-rate losses experienced by the scanner due to all (or a high percentage) of the radiotracer being present in the field of view (FoV) of the scanner during the initial phases of imaging (where the arterial input function—AIF—is derived). This dead-time effect may adversely influence the reconstructed images and subsequently the calculated perfusion parameters resulting from PET kinetic modeling. Reducing excessive count rates and hence dead-time effects can be achieved either by a limit on the activity injected as a bolus or by performing a slow infusion of the radiotracer [7].

Previous work used decaying radiation source (13-ammonia and 82-rubidium) phantoms to identify the dynamic range of the PET system and also performed MPI in a cohort of 21 patients [8]. Their results proposed a weight-based administration approach (9 ± 1.5 MBq/kg) in order to limit the prompt count rates and dead-time losses (in turn the amount of activity) required for good image quality and reliable perfusion calculation.

Recently the group also performed decaying phantom experiments on 10 modern PET-CT scanners using either 1100 MBq of [^13^N]NH_3_ or 3000 MBq of [^82^Rb]Cl [9]. Their results identified a range of scanner-dependent performance metrics to avoid bias in quantification of activity concentration in reconstructed images, such as limiting of maximum singles count rates to between 22 and 64 Mcps and also recommended weight-based injected activities in the region of 3–14 MBq/kg. They further showed the clinical effect of using 2 different weight-based administrations of [^82^Rb]Cl (2 and 10 MBq/kg, injections of 227 and 1022 MBq respectively) to the same patient, demonstrating underestimation of the peak blood and myocardium activities on the higher injected activity scan and also an overestimation of the quantified perfusion [9]. Other work has noted that although weight-based administrations may represent an oversimplification, it is a useful starting point in using phantom data to explore strategies to count-rate losses on clinical acquisitions [10].

Similar work has determined that detector blocks are less saturated with 1110 MBq infusion of [^82^Rb]Cl rather than their previous standard protocol of 1480 MBq. They noted detector block saturation in 33/217 cases, although for infusions of 1110 MBq, 99% of scans presented without saturation effects [11]. Other work using [^13^N]NH_3_ attempted to correct for underestimation in dead-time-affected input functions using phantom studies, noting an 8.9% overestimation of perfusion in 4 patients [12]. Recent attempts have been made to optimize injected activity to 11 MBq/kg (800 MBq) in [^15^O]H_2_O studies; however, these studies focused on brain imaging where lower count rates are expected [13].

Previous studies characterized the performance of simultaneous PET-MR scanners at count rates specific to the NEMA performance test or during clinical FDG imaging [14–16]. Although MPI studies have been performed on PET-MR systems using relatively low injected activities (i.e. 400 MBq 15-oxygen [17], 214 MBq [^13^N]NH_3_ [18], 396 ± 26 MBq [^13^N]NH_3_ [19] and 259–296 MBq 18F-flurpiridaz [20]), there are as yet no detailed studies of the performance characteristics of these systems at count rates expected during MPI with traditional perfusion radiotracers. Use of these tracers introduces added considerations in PET-MRI imaging, as the field of view is typically larger and ring diameter typically smaller than traditional PET-CT systems. These factors lead to higher sensitivity and hence higher count rates presenting challenges on the preservation of count-rate linearity. Further potential considerations involve increased scatter due to the presence of MR receiving coils [21].

We aim to investigate the performance of a PET-MR scanner at high count rates encountered during MPI studies using a decaying-source experiment to examine count-rate effects on quantitative accuracy, and also to investigate count-rate effects on quantification of perfusion values using a cardiac perfusion phantom to experimentally simulate clinical MPI studies.

## Methods

### Decaying-source experiment

In order to observe the count-rate response of the scanner at high activities, a decaying phantom experiment was performed. An initial activity of 1160 MBq of 11-carbon was placed in the myocardial compartment of an anthropomorphic torso phantom (phantom model ECT/TOR/P, cardiac insert model ECT/CAR/I, both Data Spectrum Corporation, Durham, USA), which represents the upper torso of average-to-large male and female patients. Two fillable defects (3.8 ml each, filled with water) were fixed in position in the myocardial chamber in the septal and lateral walls in order to evaluate if defects could be viewed over the range of activities imaged. The volume of the myocardial chamber with 2 defects attached was 110 ml, giving an activity concentration of 10.54 MBq/ml at the beginning of the scan. All other compartments of the phantom were filled with water to provide a scattering environment similar to a clinical situation.

PET scanning was performed on a 3T Siemens Biograph mMR scanner (Siemens Healthcare GmbH, Erlangen, Germany). CT images were employed for attenuation correction purposes rather than the inbuilt MR-based routine. CT scanning (140 kV, 100 mA, 0.5-s rotation, 40-mm collimation) was performed on a PET-CT scanner (GE Discovery 710, GE Healthcare, Waukesha, USA), and the resulting CT images were registered to the MR-based attenuation map using a rigid registration algorithm (Niftyreg software, University College London, UK). The registered CT data were converted to attenuation values at 511 keV using a calibration curve from the PET-CT scanner and used on the PET-MR scanner for attenuation correction of PET emission sinograms.

PET list-mode data was acquired over a 100-min acquisition. List-mode data was rebinned into 5-s frames (a frame time typically used in PET MPI) with a 2-min gap between each frame to reduce data storage requirements. Sinograms were reconstructed on the scanner front-end (OSEM 3 iterations, 21 subsets, 4-mm post-smoothing filter, 344 × 344 image matrix). All corrections for decay, dead time, scatter and randoms were utilized as implemented on the scanner.

### Cardiac perfusion phantom

In order to experimentally simulate a clinical MPI study using simultaneous PET and MR imaging, we used an in-house designed and built myocardial perfusion phantom, the construction and operation of which has previously been described in detail [22, 23]. Briefly, water is pumped through an MR-safe myocardial perfusion phantom placed in the PET-MR scanner. The phantom is representative of the large thoracic vessels and of the heart of a 60-kg body weight subject. It is composed of 4 cardiac chambers (120 ml each) and associated thoracic vessels (aorta, pulmonary artery, pulmonary vein, vena cava). Supporting precision pumping and monitoring mechanisms control the transport of water through the phantom. Myocardial perfusion is controlled in real time by continuously sampling the flow rate by means of high-precision digital flow meters (Atrato, Titan, Sherborne, UK) and providing re-adjustment of the rotation speed of roller pumps through a feedback mechanism. True perfusion values were obtained by means of measurements of the distribution volume for the radioactive tracer and gadolinium-based contrast agent (GBCA) and dividing the flow rate by this value. All controls are handled remotely from a custom-written LabVIEW application (LabVIEW Professional Development System 2014, National Instruments, Austin TX, USA) running on a dedicated workstation and remotely controlled using an iPad application (Dashboard for LabVIEW, National Instruments, Austin TX, USA). We utilized a non-recirculating model; therefor, after injection, no radiotracer or GBCA re-entered the system after transit through the phantom.

The MR perfusion sequence consisted of a clinically utilized protocol, namely a 2D TurboFLASH saturation recovery gradient echo sequence (TE = 1 ms, TR = 164 ms, Flip angle = 10°, slice thickness = 8 mm, pixel spacing = 1.875 mm, matrix size 144 × 192 voxels, with temporal resolution of 1 image per cardiac beat). MR images were acquired in a single transverse plane identified by markings on the phantom, the locations of which correspond to a known dispersion volume for the GBCA and radiotracer. Cardiac output flow rate was set to 3 l/min, with true myocardial perfusion rates (*P*_*T*_) set to 3 ml/g/min. Total PET-MR scan time for each value of *P*_*T*_ was 4 min. We utilized a dual-bolus injection technique developed by our group in order to avoid signal saturation effects [24], whereby a prebolus of GBCA is injected via a contrast injector with concentration of 0.0075 mmol/kg, followed 30 s later by the main GBCA bolus of 0.075 mmol/kg simultaneously with the radiotracer. The prebolus is free from saturation and T2* effects (due to the lower concentration) and is used in perfusion calculations as an arterial input function (AIF). All injections to the phantom were performed from the contrast injector at a rate of 4 ml/s.

In order to analyse the effects of high activity on the quantification of perfusion, we performed injections of the radiotracer to the perfusion phantom at a range of injected activities of [^18^F]F- (*A*_INJ_ = 252, 398, 594, 804 and 997 MBq). The radiotracer and the main GBCA bolus were preloaded into tubing connected to the vena cava of the phantom and injected simultaneously 1.5 min after the scan start time.

PET data were acquired in list mode, rebinned into 5-s frames, reconstructed on the scanner front-end (OSEM, 3 iterations, 21 subsets, 4-mm smoothing filter, 344 × 344 matrix). MR-based attenuation correction (MRAC) was performed using the standard dual-point VIBE T1-weighted Dixon sequence available on the scanner. After each experiment, each MRAC was visually inspected in order to check for any errors in tissue classification such as fat/water tissue inversion, which can influence the linear attenuation coefficients applied to the PET emission data [25].

The terms ‘flow’ and ‘perfusion’ have been used interchangeably in both PET and MR literature. Owing to the fact that rates of liquid through our phantom were calibrated in terms of ml/g/min (i.e. units of perfusion) and *K*_1_ values from PET kinetic modeling were in the same units, we opt to keep consistency with terminology and use the term ‘perfusion’ rather than ‘flow’ (i.e. units of ml/min).

### Image analysis

#### Decaying-source phantom

Reconstructed images of the decaying phantom experiment were analysed in PMOD (v3.7, PMOD Technologies, Zurich, Switzerland). An automated 25% isocontour was drawn around the myocardial wall and a time-activity curve (TAC) was derived. With known activity in the phantom (as assayed using a dose calibrator within 5% accuracy), the average activity concentration was plotted against true activity in the field of view (*A*_FOV_) of the scanner. Count-rate data (total prompts, randoms, trues, live-time fractions, etc.) for each time frame were extracted directly from a text file as part of the sinogram headers. Noise equivalent count rate (NECR) was calculated using the NEMA performance-testing equations (with delayed randoms) [26].

#### Perfusion phantom

Dynamic PET perfusion data were analysed in PMOD v3.7 to produce TAC of the aorta and myocardium compartments. MR images were analysed in OsiriX (OsiriX 64-bit, version 8.0.2, Pixmeo SARL, Geneva, Switzerland) to extract time-intensity curves (TIC). A single ROI of 1.6 cm (matching the tubing diameter) was placed over the aorta of the phantom, and ROIs of 4-cm diameter were placed over the left and right myocardial sections. Positioning of ROIs on the PET image plane corresponding to the MR image plane was determined from fusion of the dynamic 3D PET and 2D summed dynamic MR images in PMOD. ROIs were placed on PET images over the same spatial extent as the MR ROIs. PET VOIs were 8.1-mm thick (4 PET slices) in the axial dimension in order to match the slice thickness of the MR data (8 mm). All ROIs and VOIs were drawn on one set of PET and MR images (*A*_inj_ = 252 MBq), saved and then translated on to all subsequent scan data. Data for the left and right myocardial compartments were averaged to a single curve. We thus produced a set of TAC for PET data and TIC for MR data for the aorta (*C*_*A*(PET)_ and *C*_*A*(MR)_ respectively) and myocardial compartments (*C*_*M*(PET)_ and *C*_*M*(MR)_ respectively) over the range of activities administered to the phantom. *C*_*A*(MR)_ was derived from the prebolus peak of GBCA and not the main bolus. An estimate of total activity in each PET time frame was calculated by using a 10% threshold of the images via PMOD and multiplication by the VOI volume.

PET data was modeled using a one-tissue compartment model and two rate constants *K*_1_ (uptake rate constant in units of ml/g/min) and *k*_2_ (clearance rate constant in units of min^−1^) using the PKIN software module of PMOD. We assume an extraction fraction of 1.0 of [^18^F]F- due to the lack of any metabolic processes in the phantom, and thus, in this setup, *K*_1_ is entirely representative of perfusion. MR data was modeled in a similar fashion using the same single tissue compartment model in PMOD in order to provide relative measurements of MR-based perfusion that are independent of the effects of PET detector dead time. Area under the curve (AUC) for each TAC and TIC was also calculated as part of the modeling process.

## Results

### Decaying-source experiment

**Fig. 1:**
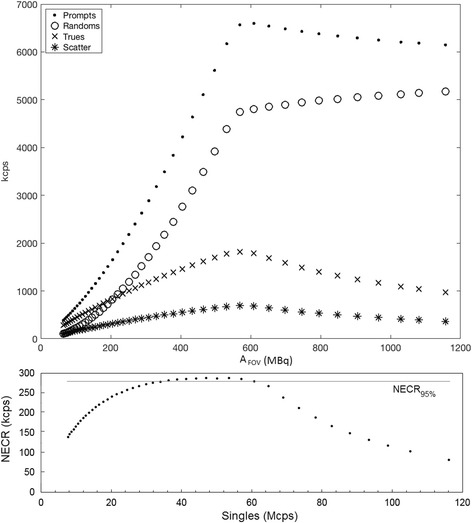
Top—Count-rate performance of the PET-MR system with activity present in the myocardial chamber of an anthropomorphic torso phantom. Of note is a discontinuity in the response at a maximum prompt rate of 6596 kcps (corresponding to a singles rate of 64.9 Mcps), above which events become affected by counting losses. Bottom—Noise equivalent count rate (NECR), with a peak rate of 286 kcps at a singles rate of 47.1 Mcps, and NECR_95%_ of 34.1 Mcps

**Fig. 2:**
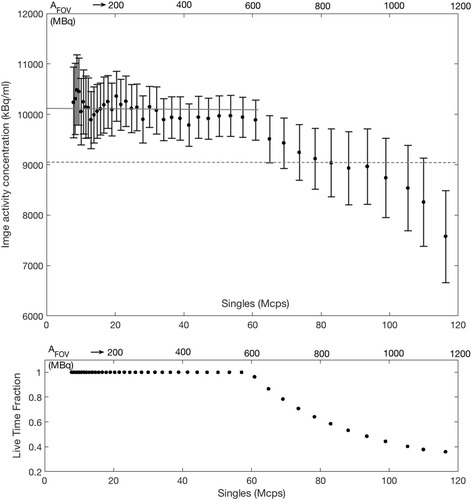
Top—The mean activity concentration of the myocardial compartment of the phantom plotted against *A*_FOV_ and singles rate showing evidence of count-rate losses above approximately 60 Mcps. The solid grey line shows the average activity concentration at count rates < 60 Mcps. The dashed line shows 10% bias from this average activity concentration reached at singles rates > 60 Mcps. Error bars represent 1 standard deviation of the mean activity concentration of the volume of interest. Bottom—Scanner live-time fraction against *A*_FOV_ and singles rate averaged over each 5-s frame showing the effect of high count rates on the ability to process true coincidence events

**Fig. 3:**
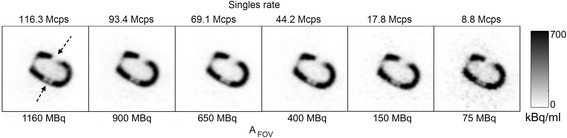
Single reconstructed transaxial slice from a 5-s frame of the decaying phantom over a range of activities and singles rates showing the locations of the 2 fixed defects placed in the myocardial compartment. The defects showed the same spatial extent over all imaged activities. All images are shown with the same window width and level

Figure 1 details the count-rate capabilities of the PET system in relation to the activity present in the myocardial section of the torso phantom. Of note in the figure is a sharp change in scanner response at a prompts rate of 6596 kcps, corresponding to a maximum singles rate over the entire scanner of 64.9 Mcps (64,954 kcps). There is a wide plateau in the NECR response (NECR_max_ = 286 kcps at 47.1 Mcps), and the plateau range calculated from 95% of the maximum (NECR_95%_) covers a range of singles rates between 34.1 and 60.9 Mcps. A lower count rate of 34.1 Mcps (corresponding to *A*_FOV_ = 305 MBq) would likely yield similar image quality. The sharp change is also evident on observing the image-based activity concentration (Fig. 2) from the reconstructed images. The point at which bias in the reconstructed images deviates from 10% of the average activity concentration at lower activities (i.e. not affected by dead time) can also be noted at 78.5 Mcps. The relationship to the live-time fraction (the fraction of events successfully detected) is also shown in Fig. 2, which details the influence of the higher count rates on the processing capability of the scanner. Figure 3 shows that despite the high count rates affecting the quantitative accuracy of the reconstructed images at high *A*_FOV_, the 5-s frames are of good visual quality and can clearly discern the defects that were inserted into the phantom at all count rates and levels of activity.

### Perfusion phantom experiments

**Fig. 4:**
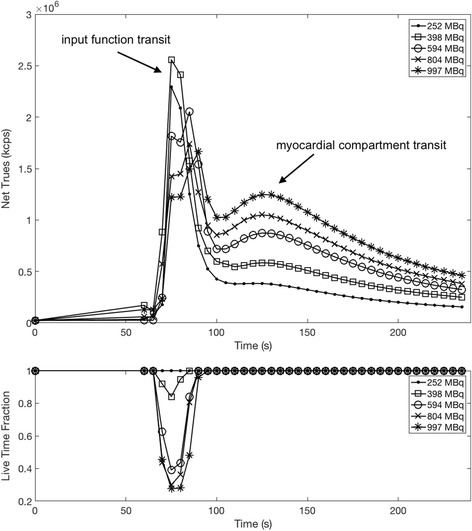
Top—Net trues and prompt rates from each injected activity experiment performed with the cardiac perfusion phantom. Of note is that above a net trues rate of approximately 2.6 × 10^6^ (corresponding to *A*_INJ_ = 398 MBq), the trues rate at peak input function actually begins to decrease due to the decrease in live-time fraction

**Fig. 5:**
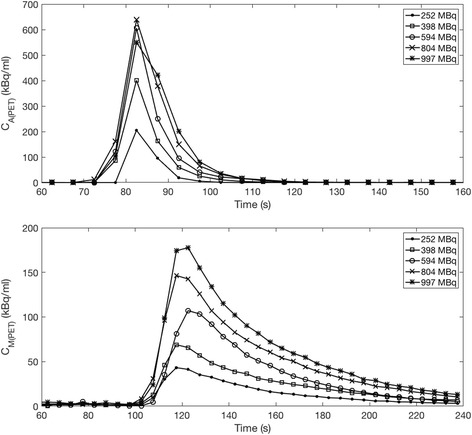
Time-activity curves resulting from image analysis with increasing activity administrations to the phantom. Top: *C*_*A*(PET)_, and bottom: *C*_*M*(PET)_, showing input and washout of the radiotracer. *C*_*A*(PET)_ for 804 and 997 MBq injected activities do not increase at the same rate as the myocardial TAC suggesting the reconstructed images are affected by count losses

The perfusion phantom experiments detail the first-pass transit of the radiotracer and GBCA through the phantom. The net number of uncorrected true coincidences over the transit of the tracer corresponding to the input function and the later transit through the myocardial compartment can be observed in Fig. 4. Peak *C*_*A*(PET)_ for *A*_INJ_ = 594, 804 and 997 MBq can be seen to be affected by count losses with increasing activity, corresponding to decreasing live-time fractions. Later transit through the myocardial compartments is unaffected with a live-time fraction of 1 for all activities. VOIs from reconstructed dynamic images observed in Fig. 5 show that peak heights of *C*_*A*(PET)_ for *A*_INJ_ = 804 and 997 MBq are reduced below the peak height for *A*_INJ_ = 594 MBq. Due to no losses post-reconstruction (a live-time fraction of 1), the increase in unaffected *C*_*M*(PET)_ with increasing *A*_INJ_ can also be observed. At peak input function, estimation of total activity in each PET frame for all activities consistently detailed that > 85% of the radiotracer was present in the FoV for each injected activity of the perfusion phantom. Using this calculation, we can assume that for each *A*_INJ_, at least 85% of each injection was present at peak input function.

**Fig. 6:**
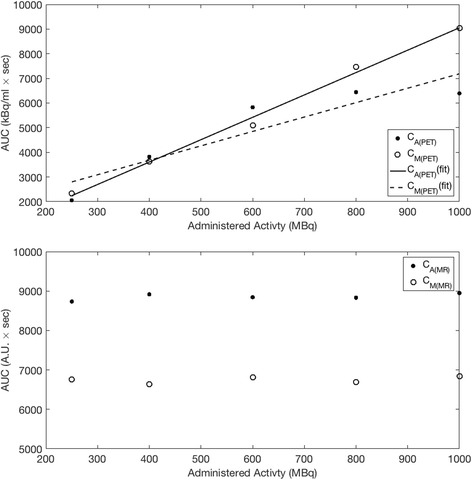
Area under the curves for (top) *C*_*A*(PET)_, *C*_*M*(PET)_, (bottom) *C*_*A*(MR)_ and *C*_*M*(MR)_. AUC increase linearly for *C*_*M*(PET)_ up to *A*_INJ_ = 997 MBq (*R* ^2^ = 0.99), whereas the effect of count losses is observed for *C*_*A*(PET)_ (*R* ^2^ = 0.72). Repeatability measurements are shown by *C*_*A*(MR)_ and *C*_*M*(MR)_

In relation to kinetic modeling, the area under the curve (AUC) of C_*A*(PET)_ is of high importance in the determination of *K*_1_ and *k*_2_ parameters. The AUC of *C*_*A*(MR)_ increases linearly (*R* ^2^ = 0.99), whereas the AUC of *C*_*A*(PET)_ for *A*_INJ_ = 804 MBq and *A*_INJ_ = 997 MBq are shown to deviate from the linear relationship derived (*R* ^2^ = 0.72) from the lower values of *A*_INJ_ (Fig. 6). In comparison, AUC of *C*_*A*(MR)_ and *C*_*M*(MR)_ show a good level of repeatability, with a constant value over all 5 datasets. A summary of the calculated perfusion values is shown in Table 1, whereby the overestimation of PET *K*_1_ can be observed where *A*_INJ_ > 594 MBq. *K*_1_ calculated from MR data shows a consistent value with low standard deviation over all datasets.

**Table 1:** Resulting perfusion parameters from phantom experiments with increasing *A*_INJ_ showing an overestimation of *K*_1_ resulting from increased areas underneath *C*_*M*(PET)_ and decreasing areas of *C*_*A*(PET)_. MRI perfusion estimations show consistent values at all activities

	PET		MRI	
*A*_INJ_	*K*_1_ (ml/g/min)	*k*_2_ (1/min)	*K*_1_ (ml/g/min)	*k*_2_ (1/min)
252	2.83	1.47	1.40	1.63
398	2.91	1.45	1.43	1.79
594	2.80	1.61	1.44	1.51
804	3.60	1.90	1.38	1.73
997	3.92	1.78	1.48	1.75

## Discussion

This work describes the effect of high count rates on the quantification of cardiac perfusion using standardized experimental simulations in an mMR PET-MR system. We have shown with a decaying-source experiment that above a total singles rate of approximately 64.9 Mcps (specific for our setup), data is subject to increasing levels of count loss. A distinct change is observed in the scanner response at 64.9 Mcps (Fig. 1). This point also corresponds to a change in recovered activity concentration, showing that at singles rates above 64.9 Mcps, quantification of images begin to be affected by count losses. For this phantom setup, a 5% reduction in NECR (NECR_95%_) can be achieved with a singles rate of 34.1 Mcps and a 10% reduction in NECR with 24.7 Mcps. Figure 2 details that a 10% bias in image activity concentration is reached in the reconstructed images between singles rates of 78.2 and 82.9 Mcps; however, bias in the images can be avoided at singles rates lower than 60 Mcps. Response in the image activity concentration in PET-CT systems typically shows an exponential increase with decreasing activity rather than a sharp change at a single activity point as observed in this work, the cause of which may be related to the avalanche photo-diode detection system on the PET-MR system rather than photomultiplier tubes on traditional PET-CT systems. We opted to choose this water-filled torso phantom with cardiac insert in order to provide a more realistic geometrical setup for scatter and tracer distribution, rather than the standard NEMA phantom used during standardized performance testing of the system. Typical NEMA measurements do not include scanner performance as a result of a bolus of a high activity radiotracer.

Our value of the maximum singles rate from the decaying-source experiment is in line with that noted by Renaud et al. [9] of 64 Mcps in their survey of PET-CT systems. Of note is that their value was determined on a scanner with a similar FoV (22.1 cm) to the mMR system (25.8 cm). Activity in the field of view between 745 and 790 MBq leading to a bias of 10% is also comparable to their work for a similar PET scanner using a similar phantom setup.

Although this phantom is a clinically unrealistic scenario for the calculation of myocardial perfusion due to a lack of redistribution of the tracer, it provides the ability to measure metrics for evaluating the count-rate performance of PET systems under a semi-clinical distribution of a radiotracer and a range of count rates observed during a clinical acquisition. Although the reconstructed images demonstrate quantitation issues at singles rates above 64.9 Mcps (Fig. 2), the fixed defects could be clearly observed in the same image regions regardless of count rate and activity present in the source (Fig. 3). Thus, the main issues with cardiac imaging relate to aspects of quantification of the PET activity concentration and thus calculation of perfusion. Our results on maximum permissible count rates differed from those provided by Delso et al. [14], as the trues rate from our experiment was linear up to 1700 kcps, rather than 700 kcps. We also noted a higher NECR_max_; however, of note is that our setup was not the standardized NEMA performance measurement as reported by Delso, and as such, our results are not directly comparable.

We used 11-carbon in the phantom rather than 15-oxygen or 13-ammonia primarily due to radiation safety aspects to the operator with filling the phantom, which would have required a much higher initial activity of a shorter-lived isotope. However, other tracers used in MPI would produce similar count rates due to similar positron branching ratios (11-carbon 99.8%, 15-oxygen 99.9%, 13-ammonia 99.8%, 82-rubidium 95%, 18-fluorine 96.9% [27]).

In addition to the decaying-source phantom, we utilized a perfusion phantom model to evaluate effects of high count rates in a semi-clinical setup. Previous work by our group has detailed a high level of repeatability using the phantom, showing consistent PET and MR perfusion calculations with a manually set perfusion rate [23]. In the work detailed here, MR perfusion was not calculated using a specific MR semi-quantification method to ensure that TAC and TIC data were analysed consistently using the same procedure. All factors (i.e. image acquisition, geometry, injection timings) for each scan were consistent. We have also previously demonstrated that PET quantification is unaffected by the presence of MR contrast agents at low concentrations [28].

Although we used MRAC for the perfusion phantom to mimic a clinical scenario, differences of 15% between the linear attenuation coefficients of MRAC and CTAC have been noted for simple water-filled phantoms [28], which would have a global scaling effect on all PET activity concentration curves.

Figure 4 details the rate of true events (i.e. events that contribute directly to the PET signal) and corresponding prompt rates for each level of activity injected into the phantom (*A*_INJ_). The trues rates of the myocardial transit phase show the expected increases with higher activity used and are unaffected by count losses as indicated by the live-time fraction. It should be noted that as in clinical studies, the amount of activity in the field of view for perfusion phantom simulations changes rapidly over time, and therefor, of primary interest to our work is the peak activity during the input phase.

Activity concentrations from the aorta and myocardium compartments (*C*_*A*(PET)_ and *C*_*M*(PET)_ respectively) are shown in Fig. 5, and it can be noted that for *C*_*A*(PET)_ for *A*_INJ_ = 804 and 997 MBq, the curves show reduction of the peak height of response. Although net trues for *A*_INJ_ = 594 show effects of high count-rate losses in Fig. 4, *C*_*A*(PET)_, conversion to activity concentration in Fig. 5 shows that this may be to some extent recoverable or shows only a minor effect potentially as the activity injected (594 MBq) is close to the 570 MBq point identified from the decaying-source experiment. It should be noted that, owing to dispersion of the radiotracer, not all of the radiotracer injected is present in the scanner FOV simultaneously.

Calculation of the area under the curve (AUC) for each *C*_*A*(PET)_ and *C*_*M*(PET)_ shown in Fig. 6 demonstrates that *C*_*M*(PET)_ increases linearly over all tested activities (*R* ^2^ = 0.99) while *C*_*A*(PET)_ is linear only up to *A*_INJ_ = 594 MBq with count-rate losses above this point affecting linearity. Calculated values of perfusion using the same methodology for PET and MR data (Table 1) also show that for *A*_INJ_ > 594 MBq, increases in *K*_1_ can be attributed to a decreased *C*_*A*(PET)_ and increased *C*_*M*(PET)_ as evident in Fig. 4. Consistent values for *K*_1_ from the MR curves show that good repeatability can be obtained by use of the phantom. Direct comparison of PET and MR *K*_1_ values should be avoided, as MR values were calculated using a PET kinetic model from the prebolus peak and using signal intensity values as PET activity concentration.

It should be noted that the scattering scenarios between the perfusion phantom and the decaying-source phantom are different, given that the perfusion phantom is not surrounded by water. Therefor, the maximum trues rates between the setups will be different based on the lower number of scatter and randoms in the decaying-source experiment. However, even with this difference of phantom environments, insights can be gained into a limiting value of count rate for accurate quantification. Further limitations of the phantom model compared to a clinical situation are that the phantom represents an oversimplification of the cardiovascular system and cannot detail true myocardial diffusion or intracellular uptake of PET radiotracers. It is also unable to reproduce the multiple sources of image artifacts in clinical PET-MR (such as respiratory or cardiac motion). Despite these seemingly big limitations, the setup provides an environment free from these potentially confounding effects, allowing analysis to focus only on the assessment of the perfusion dynamics within the cardiac compartments, and the potential to repeatedly examine count-rate capability for determination of perfusion accuracy. Furthermore, it allows technical development of imaging techniques related to myocardial perfusion without the need to expose patients to ionizing radiation or contrast agents. In this respect, the standardized and repeatable setup is a considerable advantage over routine clinical studies for the evaluation of the count-rate losses due to the effects of high activity in the FOV.

Count rates in clinical studies are likely to be lower than those described in the perfusion phantom experiments due to the different scattering geometry of a patient, dispersion of radiotracer, and also the time taken to transit the pulmonary circulation and reach the left ventricle before being measured as part of the input function. Therefor, the singles rates provided by this work may represent an upper maximum of clinically observed count rates and will be compared to clinical studies in the future. Refinement to the acquisition and list-mode rebinning protocol should be investigated using the perfusion phantom (and in patients) using methods such as that described by Kolthammer et al. to optimize the frame time in relation to variables such as the infusion duration, scan time and total activity [29]. For simplicity, we used a constant time frame over the entire study, in a similar fashion to previous work [9].

Our findings indicate that singles rates above 64.9 Mcps will affect the quantitation of resulting PET images, potentially impacting the arterial input functions and thus calculation of perfusion. However, patient studies should be performed in order to compare the count rates observed on the phantom to those found during clinical acquisitions and also to examine the effects of lower count rates on image quality.

## Conclusions

Our work on a decaying-source phantom has identified that a singles rate above 64.9 Mcps during the first pass of the radiotracer on the mMR system affects the quantitation of image-based arterial input functions, and a singles rate between 78.2 and 82.9 Mcps leads to a 10% bias in recovered image activity concentration. NECR_max_ was 286 kcps at 47.1 Mcps, and NECR_95%_ was observed from a singles rate of 34.1 Mcps up to 60.1 Mcps. Our cardiac perfusion phantom with a perfusion rate of 3 ml/min/g showed count losses with increasing singles rates above 64.9 Mcps, corresponding to an injected activity to the phantom of 600 MBq and higher. Above this count rate, at least 55% of the events were lost and quantification of perfusion was severely affected. Quantification of perfusion values with singles rates above 64.9 Mcps on this system may be compromised and should be avoided where possible. Validation studies in large animals could be performed to verify the results of the phantom experiments, as well as to investigate any potential effects on image quality.
